# Effect of *Nigella sativa* L. Seed on the Kidney of Monosodium Glutamate Challenged Rats

**DOI:** 10.3389/fphar.2022.789988

**Published:** 2022-06-14

**Authors:** Mahmoud Abd-Elkareem, Mahmoud Soliman, Mokhless A. M. Abd El-Rahman, Nasser S. Abou Khalil

**Affiliations:** ^1^ Department of Cell and Histology, Faculty of Veterinary Medicine, Assiut University, Assiut, Egypt; ^2^ Department of Pathology and Clinical Pathology, Faculty of Veterinary Medicine, Assiut University, Assiut, Egypt; ^3^ Department of Food Science and Technology, Faculty of Agriculture, Assiut University, Assiut, Egypt; ^4^ Department of Medical Physiology, Faculty of Medicine, Assiut University, Assiut, Egypt

**Keywords:** monosodium glutamate, Nigella sativa, kidney, antioxidants, apoptosis

## Abstract

Monosodium glutamate (MSG) consumption is responsible for a wide spectrum of health hazards including nephrotoxicity. The search for phytochemical strategies having broad safety profile to counter MSG toxicity is worthwhile. *Nigella sativa* L. seed (NSS) is very promising in this regard owing to its antioxidant and cytoprotective nature. Therefore, we attempted to investigate the potential protective effect of NSS on MSG-induced renal toxicity in rats. To accomplish this objective, fifteen adult Wistar albino rats were randomly and equally divided into three groups for 21 days: the control group received no treatment, MSG group supplemented with MSG at a dose of 30 g/kg feed, and MSG + NSS group supplemented with MSG at the same previous dose in conjugation with NSS at a dose of 30 g/kg feed. MSG and its combination with NSS failed to cause any significant difference in the kidney function parameters in comparison with the control. A significant elevation in lipid peroxides (LPO) level, glutathione-S-transferase activity and total antioxidant capacity (TAC) and a significant reduction in superoxide dismutase activity were found in MSG group. LPO level and TAC in MSG intoxicated rats significantly normalized by NSS ingestion. NO level showed absence of significant difference among all experimental groups. MSG elicited histopathological lesions such as decreased glycoprotein content and fibrosis however, NSS succeeded in enhancing all these features. MSG group showed positive glutathione reductase and superoxide dismutase 2 immuno-expression whereas, MSG + NSS group showed weak immunostaining. A significant increase in the number of apoptotic cells was observed in MSG group compared to the control. On the other hand, MSG + NSS group exhibited a significant decrease in the number of apoptotic cells. NSS mitigated MSG-induced renal impairments by ameliorating oxidative stress and exerting anti-apoptotic effect.

## Introduction

Although commercial foods are time and energy saving, they adversely impacted the health of consumers as their manufacturing needs the addition of food additives to prevent the growth of microorganisms that cause spoilage or foodborne illness and to increase the consumer acceptability (Miyaki et al., 2016; Pasca et al., 2018; Chakraborty, 2019). In this regard, monosodium glutamate (MSG), a sodium derivative of l-glutamate, is still globally incorporated in a broad spectrum of food preparations (Hajihasani et al., 2020) for its umami taste and flavor enhancement properties (Henry-Unaeze, 2017). Many types of food contain MSG, for example, processed meat, soup bases, flavored snacks, spices, gelatin containing substances, and bodybuilding protein powder (Lavine, 2007; Populin et al., 2007). There are two different research directions in the literature regarding its safety as a food additive. Food safety regulatory agencies generally consider MSG consumption is not associated with health hazard issues (Zanfirescu et al., 2019) based on the fact that glutamate does not passively cross the cellular membranes, and is completely metabolized by enterocytes as an energy substrate (Henry-Unaeze, 2017). Owing to the plenty of polyunsaturated fatty acids in the renal lipid profile, the kidney is supposed to be a high-ranked organ in its vulnerability to free radical attack (Kubo et al., 1997). During the last decade, it became evident that the long-term administration of MSG has deleterious effects on the kidneys by disturbing redox homeostasis, and inducing lipid peroxidation and histo-architecture lesions (Sharma, 2015).

Some scholarly articles highlighted the protective effect of NSS against MSG-induced renal toxicity (Moussa and Al Mulhim, 2013; Yousif et al., 2016) showing a marked improvement in the histopathological and histochemical features along with inflammatory mediators without measuring redox, apoptotic, and kidney function parameters. This gives a driving force for our study to explore these mechanistic tools in mediating the reno-protective effect of NSS using an experimental rat model.

Medicinal plants represent an indispensable cornerstone in curing many diseases since several centuries being superior to the traditional medicine in their wide safety and availability (Ahmad et al., 2013). Among them having highly promising health benefits, *Nigella sativa* L. also called black cumin or black seeds. In Islamic literature, it is considered to be one of the greatest forms of healing medicine as it was mentioned that the black seed is the remedy for all diseases except death in one of the Prophetic hadiths. It is also recommended to be used on regular basis in Tibb-e-Nabwi (Prophetic Medicine) (Al-Bukhari and Sahi, 1976). Its activities in blocking oxidative stress and apoptotic cascade along with improving renal damage biomarkers and histopathological features (Hosseinian et al., 2018; Akinwumi et al., 2020) make it highly accepted as a realistic option for fighting food-borne nephrotoxicants. The abundance of phytochemicals in NSS gives a strong driving force for numerous scientists trying to confirm its conventionally claimed use in combating renal dysfunction.

However, whether NSS could protect against MSG-induced renal toxicity and what are its renal protectant mechanisms in the front of MSG overtake are questions still needed to be answered.

## Materials and Methods

### Screening of Active Phytochemical Ingredients

NSS was provided by Imtenan Heath Shop Company, Obour City, Egypt. Phytochemical constituents of NSS were determined using gas chromatography/mass spectrophotometry (Triple Quadrupole) (GC-MS, 7,890A-5975B, Agilent Technologies, United States), coupled with fused silica DB-5 capillary column (30 m length, 0.25 mm internal diameter, and 0.25 µm film thickness). A sample from the seeds was ground. One gram of the plant powder was weighted followed by the addition of 1.5 ml of chloroform. The mixture was sonicated at 35°C for 1 min followed by centrifugation at 8,000 rpm for 15 min at 40°C. Then, the clear organic layer was withdrawn followed by injection in GC-MS. Column initial was held for 2 min at 40°C, then increased to 150°C at 10°C/min, and held for 3 min, then raised to 220°C with the same rate and held for 6 min, then raised to 280°C and held for 15 min 1 µL of the sample was injected with splitless injection mode. The carrier gas was helium with a flow rate of 0.5 ml/min for 10.9 min, then 1 ml/min for 30 min. The injector and detector temperatures were 250 and 290°C, respectively. Mass spectra were scanned in the range of 40–1,000 amu. The scan time was five scans/s. The constituents were identified by the combination of retention index data and mass spectra using the Wiley nine NIST 11 library.

### Experimental Design

Fifteen adult Wistar albino rats of both sexes, 2-3-months-old (233.820 ± 9.099 g), were allowed to acclimatize for 1 week and then randomly and equally assigned to control, MSG, and MSG + NSS groups. The control group received no treatment, while the MSG group administrated MSG (Morgan Chemical industry, Egypt, purity 99%) at a dose of 30 g/kg feed (Kazmi et al., 2017) thoroughly mixed with the ration for 21 days. MSG + NSS group administrated MSG at the same previous dose in addition to NSS, after finely crushed using an electric mill mixer, and added to the diet as a powder at a dose of 30 g/kg feed for the same period. This dose is well established to protect against various toxicants (Abd-Elkareem et al., 2021). Rats were housed at 24 ± 1°C in polypropylene cages on wood chips, exposed to a natural daily light/dark cycle, and provided a standard rat chow pellet and water *ad libitum*. The experimental protocol has fulfilled all requirements as governed by the Declaration of Helsinki and approved by the Institutional Review Board of Faculty of Medicine in Assiut University, Assiut, Egypt (Approval Number: 17300469).

### Sample Collection

After the treatment schedule, blood samples were collected from the retro-orbital plexus of each rat after a 12 h fast using heparinized microcapillary tubes. Blood samples were collected and centrifuged at 3,000 rpm for 15 min using centrifuge (V/O MEDEXPORT, N 0649, USSR) to obtain serum which was kept at −20°C for estimation of renal function and redox parameters. The kidneys were subsequently removed from the control and treated rats after they were euthanized by cervical dislocation. The kidneys were carefully dissected out and quickly fixed in 10% neutral buffered formalin to be used in histological, histochemical, and immunohistochemical studies.

### Biochemical Analysis

Urea was assessed according to the modified urease-Berthelot method (Tietz, 1990). Buffered kinetic jaffe reaction without deproteinization (Bowers and Wong, 1980) was used for the measurement of creatinine. The assay of uric acid is based on the method of modified trinder peroxidase using 3,5-dichloro-2-hydroxybenzenesulfonic acid (Barham and Trinder, 1972). Lipid peroxides (LPO) were estimated according to the method of (Ohkawa et al., 1979) using tetramethoxypropane as an external standard. Nitric oxide (NO) was measured following the procedure of (Ding et al., 1988). Briefly, phosphoric acid is quantitatively converted to a diazonium salt by reaction with nitrite in an acid solution. The diazonium salt is then coupled to naphthalene diamine dihydrochloride forming an azo dye that can be spectrophotometrically quantitated based on its absorbance at 550 nm. Glutathione-S-transferase (GST) was estimated by measuring the conjugation of one- chloro- 2,4- dinitrobenzene with reduced glutathione (Habig et al., 1974). The conjugation is accompanied by an increase in absorbance at 340 nm. The rate of increase in absorbance is directly proportional to the GST activity in the sample. Superoxide dismutase (SOD) was estimated based on the ability of the enzyme to inhibit the phenazine methosulphate-mediated reduction of nitroblue tetrazolium dye (Nishikimi et al., 1972). Total antioxidant capacity (TAC) was assessed according to the manufacturer’s instructions by a commercial colorimetric kit (Catalog number: TA 2513) obtained from the Egyptian Company for Biotechnology, Cairo, Egypt.

### Histological Examination

The formalin-fixed samples were dehydrated in ascending grades of ethanol, cleared in methyl benzoate, and then embedded in paraffin wax. Paraffin sections at 5 µm in thickness were cut and stained with the following histological stains:

1) Haematoxylin and Eosin for general histological examination (Bancroft and Gamble, 2008).
2) Periodic acid Schiff (PAS) technique for demonstration of neutral mucopolysaccharides (Abd-Elkareem et al., 2021).
3) Crossmon’s trichrome technique to stain collagen fibers (Crossmon, 1937).

Paraffin sections were examined by an Olympus BX51 microscope and the photos were taken by an Olympus DP72 camera adapted into the microscope. The histological evaluation was performed in a blind fashion on coded samples, and a comparison was made with the sections from the control group.

### Immunohistochemistry of Glutathione Reductase and Superoxide Dismutase 2 in the Kidney

For immunohistochemical detection of glutathione reductase (GR) and superoxide dismutase 2 (SOD2) in the kidney, we used polyclonal anti-glutathione reductase and anti-superoxide dismutase 2, respectively (Chongqing Biospes Co., Ltd., China) and Power-Stain™ 1.0 Poly horseradish peroxidase (HRP) DAB Kit (Genemed Biotechnologies, Inc., 458 Carlton Ct. South San Francisco, CA 94080, U.S.A). Sections (3 to 5 μm) of paraffin-embedded tissues were dewaxed by immersing the slides in xylene two times for 15 min each; rehydrating the slides in 100, 100, 95, 80, and 70% solutions of ethanol for 5 min each; and rinsing them in PBS pH 7.4 (3 times for 5 min each). For antigen retrieval, the slides were placed in 10 mM sodium citrate buffer (pH 6.0) and heated to near-boiling (95–98°C) in a water bath for 20 min. They were then cooled for 20 min at room temperature. The sections were then rinsed in PBS pH 7.4 (3 times for 1 min each). Endogenous peroxidase was inhibited by incubating the slides in 3% hydrogen peroxide for 10 min at room temperature before washing the slides in PBS pH 7.4 (3 times for 5 min each). The slides were then incubated with the specific primary antibodies (1:100) for 1 h at room temperature. The slides were then rinsed with PBS pH 7.4 (3 times for 2 min each) and incubated for 15 min in poly HRP conjugate, the slides were then rinsed in PBS pH 7.4 (3 times for 2 min each). Visualization of the bound antibodies was carried out by adding 200 μL of mixed substrate solution and incubating the slides for 5–10 min at room temperature. A ready-to-use DAB substrate solution was prepared by adding DAB chromogen solution to DAB buffer solution and then mixing the two solutions in a 1:1 ratio. Then, the slides were rinsed with tap water to remove excess substrate solution. The sections were counterstained in Harris hematoxylin for 30 s. The sections were then dehydrated in a graded series of ethanol (95% ethanol and then twice in 100% ethanol), cleared with xylene, and mounted with DPX. All staining preparations were examined with an Olympus BX51 microscope, and photos were taken by an Olympus DP72 camera attached to the microscope.

### TUNEL Assay

Detection and quantification of apoptosis were carried out using *In Situ* Cell Death Detection Kit, Fluorescein (Sigma-Aldrich). This TUNEL technology was based on the labeling of DNA strand breaks, which formed during apoptosis as a result of the cleavage of genomic DNA. Sections (3–5 µm) of paraffin-embedded tissues were dewaxed in xylene and rehydrated through a graded series of ethanol and double distilled water. Then slides were rinsed in PBS at a pH of 7.4 (three times for 5 min each time). The slides were placed in a jar containing 100 ml 0.1 M citrate buffer, pH 6.0, and heated to near boiling (95–98°C) in a water bath for 30 min followed by cooling for 20 min at room temperature. Sections were then rinsed in PBS at a pH of 7.4 (three times for 1 min each time). TUNEL reaction mixture was prepared by adding the total volume (50 µL) of enzyme solution to 450 µL label solution to obtain 500 µL TUNEL reaction mixtures. Then mixed well to equilibrate components. Slides were rinsed three times with PBS at 15–25°C and excess fluid was drained off. Then drops of TUNEL reaction mixture were added to the samples and slides were incubated overnight in a humidified atmosphere at 37°C in the dark. Slides were rinsed three times with PBS and directly analyzed under a fluorescence microscope. The apoptotic cells were counted in 10 high power fields and were calculated by dividing the total number of apoptotic cells by the total number of cells and multiplying by 100.

### Statistical Analysis

Data were expressed as the mean ± standard error of the mean (SEM). Statistical differences between groups were identified by one-way analysis of variance (ANOVA) followed by the Duncan post hoc test. All statistical analyses were carried out using SPSS for Windows software, version 16.0 (SPSS, Inc. Chicago, IL, United States). A probability (*p*) value of <0.05 was considered statistically significant.

## Results

### Bioactive Constituents of *Nigella sativa* L. Seed

We previously reported that the gas chromatography/mass spectrophotometry of NSS revealed the presence of 27 bioactive phytochemical constituents. 9,12-octadecadienoic acid (88.40%), hexadecanoic acid (7.48%), and thymoquinone (1.5%) represented the most predominant fractions (Abd-Elkareem et al., 2021).

### Effect of *Nigella sativa* L. Seed on the Kidney Function Parameters in Monosodium Glutamate Challenged Rats

As shown in Table 1, neither MSG nor its combination with NSS caused any significant difference in the kidney function parameters compared to the control.

**TABLE 1:** Effect of *Nigella sativa* L. seed on kidney function and oxidant/antioxidant parameters in the serum of monosodium glutamate challenged rats.

Group parameter	Control	MSG	MSG + NSS	*p* Value
Urea level (mg/dl)	25.700 ± 0.817	26.083 ± 0.734	24.950 ± 0.431	0.505
Creatinine level (mg/dl)	0.608 ± 0.083	0.528 ± 0.053	0.514 ± 0.054	0.553
Uric acid level (mg/dl)	2.650 ± 0.316	2.583 ± 0.170	3.283 ± 0.310	0.171
LPO level (nmol/ml)	1.332 ± 0.091^b^	2.033 ± 0.298^a^	1.119 ± 0.075^b^	0.005
NO level (nmol/ml)	88.130 ± 6.805	111.160 ± 20.004	79.940 ± 12.353	0.288
SOD activity (U/mL)	71.597 ± 2.236^a^	62.266 ± 2.270^b^	61.190 ± 1.873^b^	0.004
GST activity (U/L)	19.900 ± 3.466^b^	37.020 ± 3.297^a^	38.760 ± 3.443^a^	0.006
TAC (mM/L)	0.520 ± 0.317^b^	1.118 ± 0.093^a^	0.629 ± 0.045^b^	0.000

MSG: monosodium glutamate; NSS: *nigella sativa* seed; LPO: lipid peroxides; NO: nitric oxide; SOD: superoxide dismutase; GST: glutathione-S-transferase; TAC: total antioxidant capacity.

Results are expressed as the mean ± SEM of five rats per group. ^a,b^ Different letters indicate significant differences at *p* < 0.05 (one-way ANOVA followed by Duncan’s post-test).

### Effect of *Nigella sativa* L. Seed on the Redox Homeostasis of Monosodium Glutamate Challenged Rats

MSG supplemented rats had an oxidant/antioxidant imbalance manifested by a significant elevation in LPO level, GST activity, and TAC along with a significant reduction in SOD activity. LPO level and TAC in MSG exposed rats returned to the normal after dietary consumption of NSS. NSS failed to change GST and SOD activities in MSG exposed rats. There was no significant difference between NO level among all experimental groups (Table 1).

### Effect of *Nigella sativa* L. Seed on the Histopathological Features of Kidney in Monosodium Glutamate Challenged Rats

**FIGURE 1:**
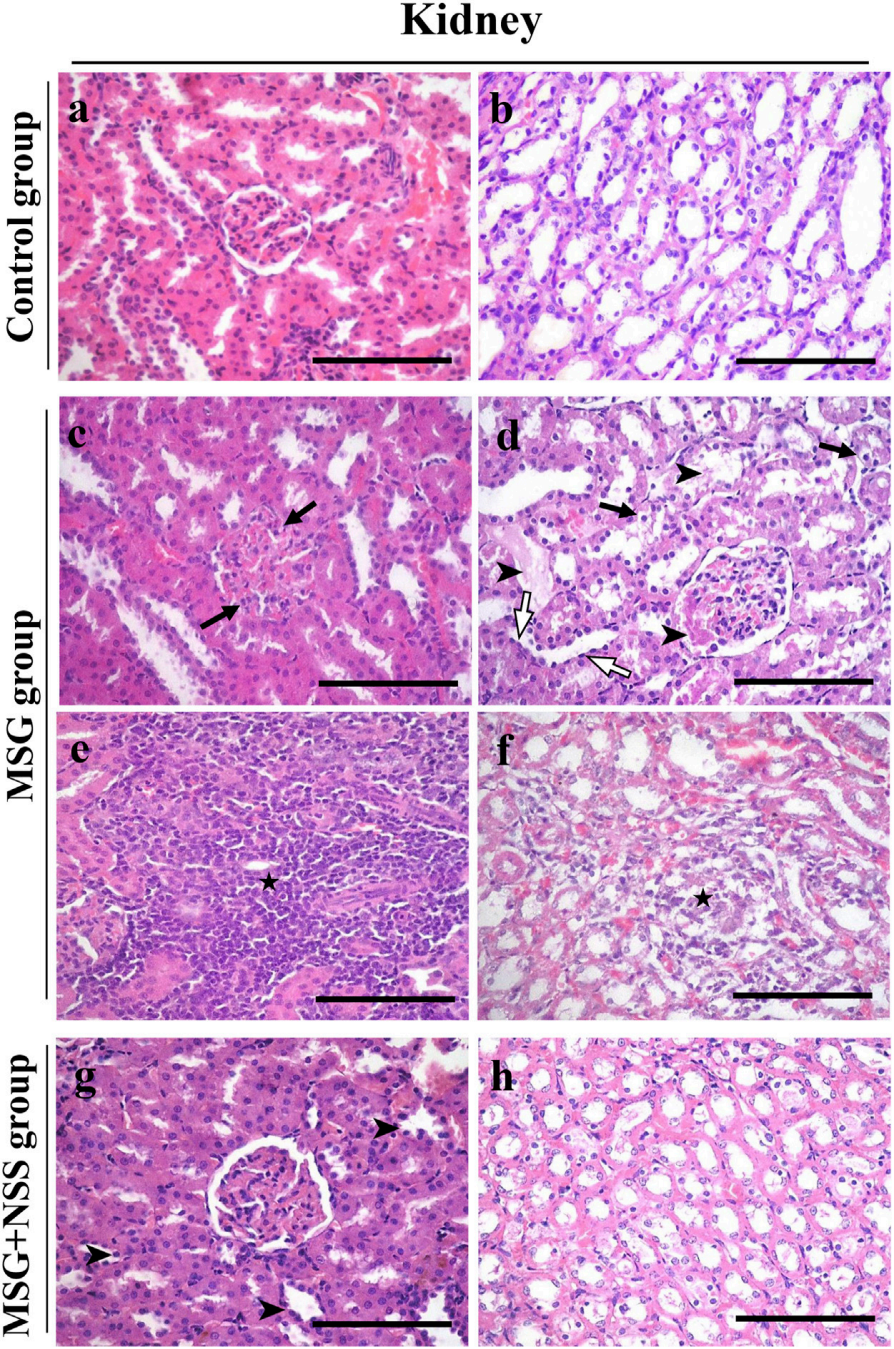
Histopathological changes of the kidney in control-, monosodium glutamate (MSG)-, and MSG + *Nigella sativa* seed (NSS)-treated groups **(A,B)** Kidney from the control group showed a normal renal cortex, containing normal glomerulus with Bowman’s space and the surrounding renal tubules **(A)**, and renal medulla **(B) (C–F)** MSG-treated group showed membranoproliferative glomerulonephritis (arrows) **(C)** leading to hyalin casts in the bowman’s space and renal tubular lumens (arrowshead) **(D)**. The renal tubules showed degeneration and necrosis and vacuolated cytoplasm (arrows) **(D)**, and flattened epithelium (white arrows) **(D)**. Mild congestion and lymphoid cell aggregation in the interstitium (star) **(E)**. The medullary region showed necrosis and lymphoid cell infiltration (star) **(F) (G,H)** Kidney from the MSG + NSS-treated group showed shortening of the tubular epithelium (arrowshead) **(G)** and minimal necrotic area in the medulla **(H)**. Hematoxylin and eosin stain. Scale bars = 50 μm.

The kidney sections of the control group showed the normal structure of the cortex containing glomerulus with surrounding renal tubules and medulla. The glomerulus had numerous capillary loops, with normal numbers of endothelial and mesangial cells. Bowman’s capsule consisted of an inner visceral, outer parietal layer and Bowman’s space in-between. The surrounding renal tubules were lined with cuboidal epithelium (Figures 1A,B). The histopathological changes in the kidney of the MSG group were defined by membranoproliferative glomerulonephritis and hyaline casts in the Bowman’s space and renal tubular lumens (Figures 1C,D). The renal tubules also showed degeneration and necrosis, shortening and flattening of the lining epithelium, and vacuolation of the cytoplasm (Figure 1D). There was mild congestion and mild to moderate lymphoid cell infiltration in the interstitial tissue (Figure 1E). Necrosis and infiltration of lymphoid cells were found in the medulla (Figure 1F). However, these renal changes were markedly attenuated following treatment with NSS, as glomerulus and renal tubules were protected from degeneration and necrosis. NSS group did not show the alterations observed in the MSG group (Figures 1G,H).

### Effect of *Nigella sativa* Seed on Glycoprotein Content and Collagen Deposition in the Kidney of Monosodium Glutamate Challenged Rats

**FIGURE 2:**
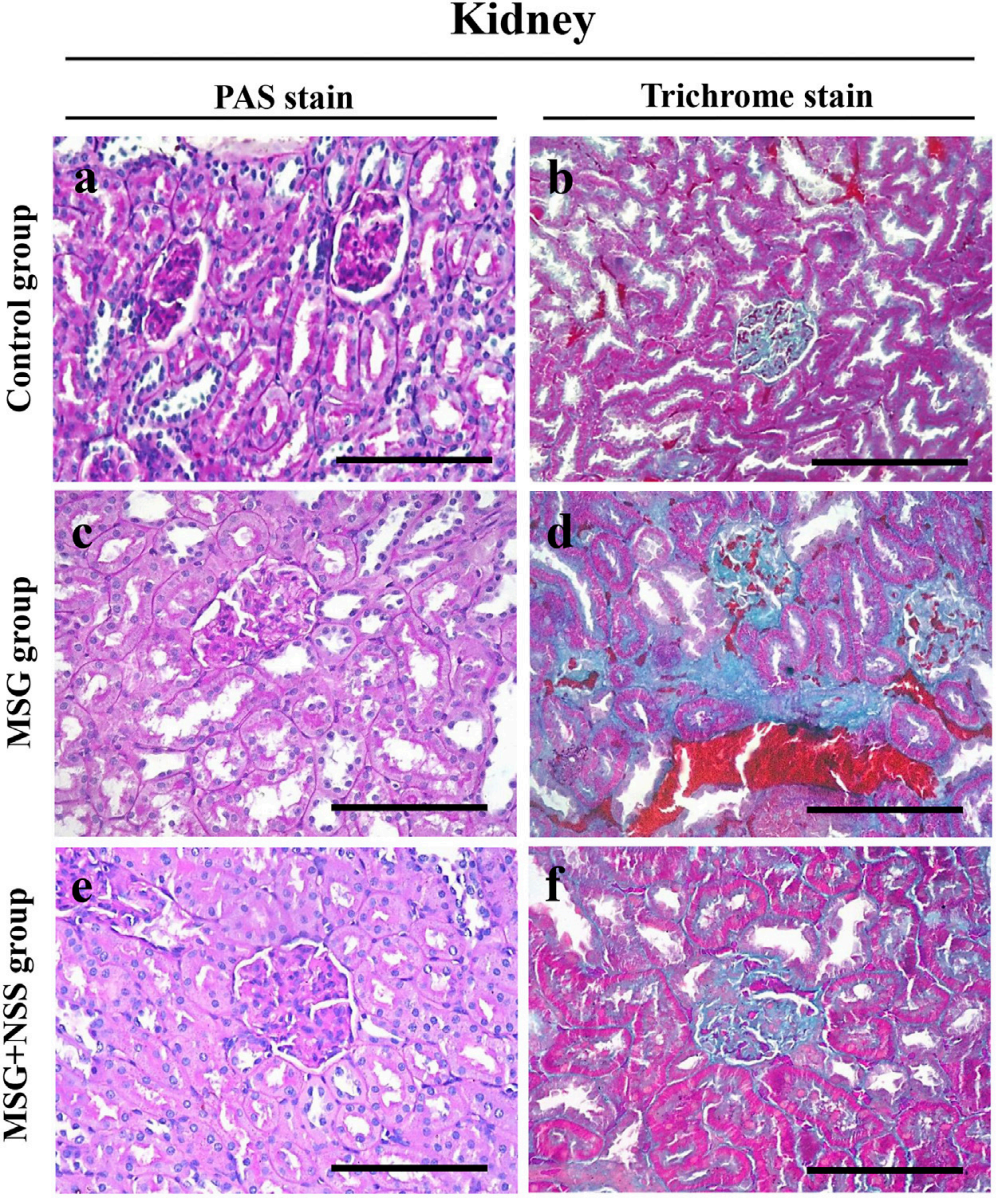
Evaluation of the glycoprotein and collagen fibers in the kidney using PAS and Crossmon’s trichrome stains, respectively, **(A,B)** Kidney from the control group showed normal glomerulus and tubules **(A)** and normal collagen fibers amount and distribution **(B) (C,D)** Kidney from the MSG-treated group showed glomerular fibrosis, loss of the tubular brush border, and deposition of intratubular protein casts **(C)** and dense collagen fibers deposition **(D) (E,F)** kidney from the MSG + NSS-treated group showed minimal glomerular and tubular damage **(E)** and improved collagen fibers deposition **(F)**. PAS and Crossmon’s trichrome stain. Scale bars in panels **(A–F)** = 50 µm.

Kidney sections from the control group showed normal glomeruli and tubules (Figure 2A). In the MSG group, there was fibrosis of the glomeruli and an increased number of mesangial cells. The renal tubules showed degeneration with loss of the brush border and intratubular protein casts (Figure 2C). However, the NSS group showed minimal changes in the kidney (Figure 2E). There was also a normal amount and distribution of collagen fibers in the kidney of the control group (Figure 2B). Dense collagen accumulation in the glomerulus and interstitial fibrosis indicated tissue fibrosis in the kidney of the MSG group (Figure 2D). NSS group showed a marked improvement in the amount and distribution of the collagen fibers (Figure 2F).

### Effect of *Nigella sativa* L. Seed on Immuno-Expression of Glutathione Reductase and Superoxide Dismutase 2 in the Kidney of Monosodium Glutamate Challenged Rats

**FIGURE 3:**
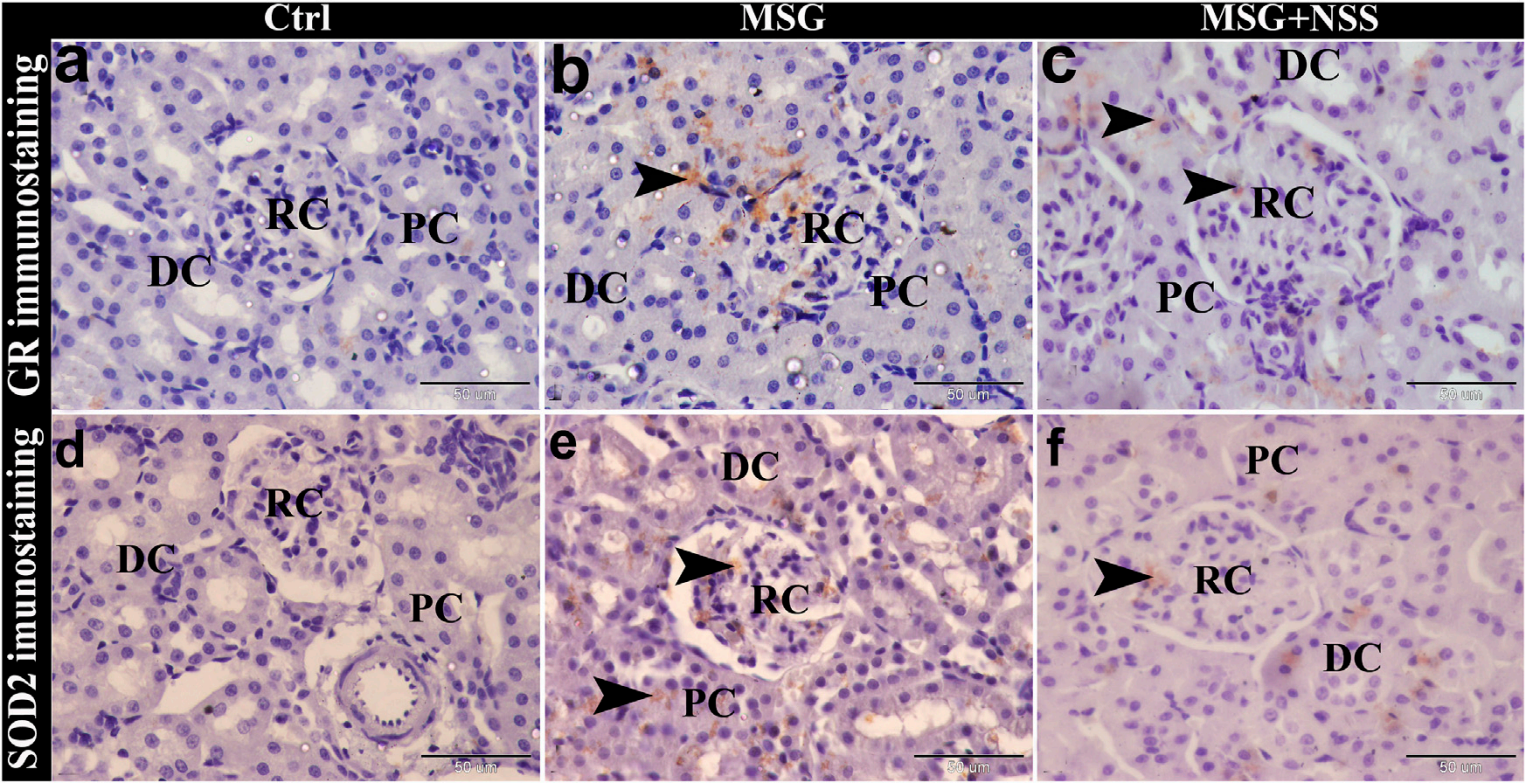
Photomicrograph of GR and SOD2 immunostaining in the kidney showing the protective effect of NSS on MSG induced renal damage. The control group (Ctrl) showed negative immunostaining of GR **(A)** and SOD2 **(D)** in the renal corpuscle (RC), proximal convoluted tubules (PC), and distal convoluted tubules (DC). MSG group showed positive immunostaining of GR **(B)** and SOD2 **(E)** (arrowheads) in the RC, PC, and DC. MSG + NSS group showed weak immunostaining of GR **(C)** and SOD2 **(F)** (arrowheads) in the RC, PC, and DC. Original magnification, ×400, scale bar = 50 μm.

GR and SOD2 immunohistochemical investigation in the kidney of the MSG group showed positive GR and SOD2 immuno-expression in some renal corpuscle, proximal convoluted tubules and distal convoluted tubules (Figure 3). Whereas GR and SOD2 immunolocalization in the MSG + NSS group showed weak GR and SOD2 immunostaining in the renal corpuscle, proximal convoluted tubules and distal convoluted tubules, and was nearly similar to the control group (Figure 3).

### Effect of *Nigella sativa* L. Seed on the Number of Apoptotic Cells in the Kidney of Monosodium Glutamate Challenged Rats

**FIGURE 4:**
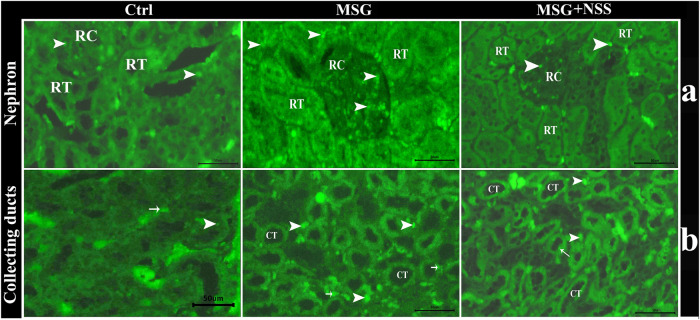
Fluorescent photomicrograph of TUNEL assay in paraffin sections in kidney. showing the protective effect of NSS on MSG induced renal damage. **(A)** renal cortex in control group (Ctrl) showed few number of apoptotic cells (arrowhead) in renal corpuscles (RC) and renal tubules (RT), renal cortex in MSG group showed high number of apoptotic cells (arrowheads) in RC and RT, and renal cortex in MSG + NSS group showed few number of apoptotic cells (arrowheads) in RC and RT. **(B)** Renal medulla in control group (Ctrl) showed few number of apoptotic cells in the collecting ducts (arrow) and in the interstitial tissue (arrowheads), renal medulla in MSG group showed high number of apoptotic cells in the collecting ducts (arrows) and in the interstitial tissue (arrowheads), renal medulla in MSG + NSS group showed few number of apoptotic cells in the collecting ducts (arrow) and in the interstitial tissue (arrowheads). Scale bars = 50 μm.

**FIGURE 5:**
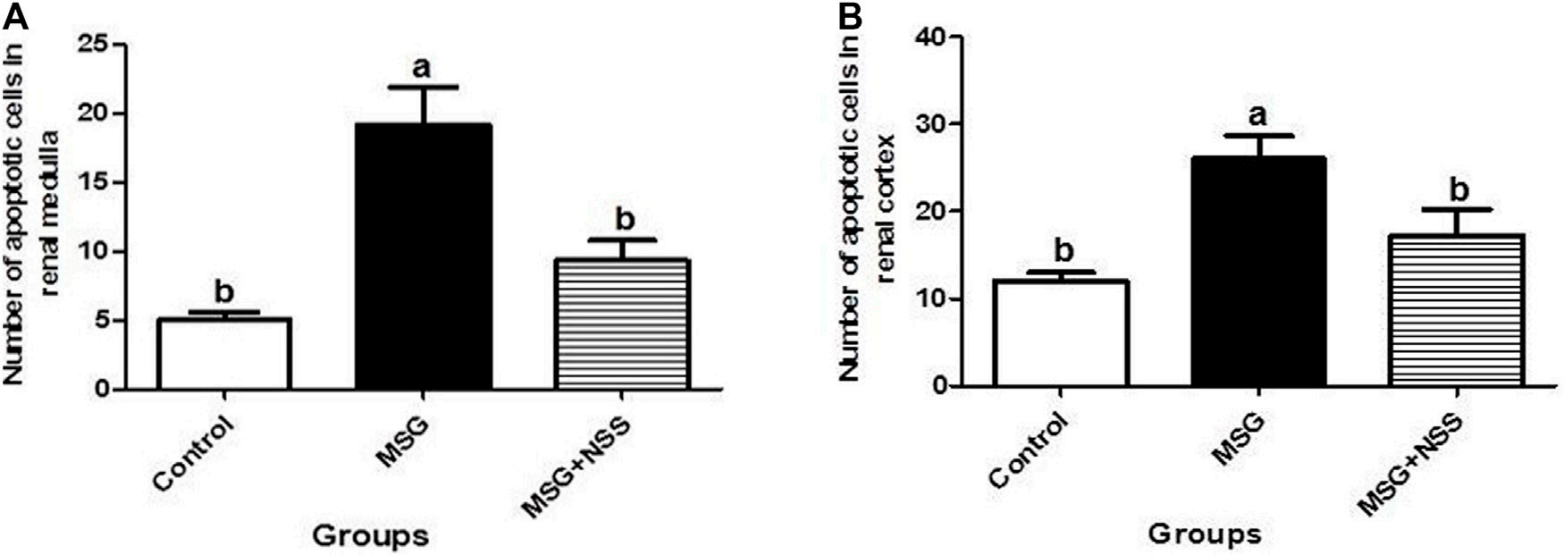
Morphometric analysis of the number of apoptotic cells in the renal medulla **(A)** and cortex **(B)**. Results are expressed as the mean ± SEM. ^a^Significantly different from the control group and ^b^significantly different from the MSG group and indicate significant differences at *p* < 0.05 (one-way ANOVA followed by Duncan’s post-test).

Using TUNEL assay in the paraffin sections of the kidney, we observed that the renal cortex (renal corpuscles and renal tubules) and the renal medulla (collecting ducts and interstitial tissue) in the MSG group showed a significant increase in the number of apoptotic cells compared to the control group (Figure 4). Whereas the kidney in the MSG + NSS group showed a significant decrease in the number of apoptotic cells which returned to the normal (Figure 5).

## Discussion

Our main findings were that MSG in rats triggered oxidative stress along with renal histochemical impairments and apoptosis. However, the current dose and duration of exposure were unable to exert any marked changes in the kidney damage biomarkers in association with the histopathological lesions. Alternatively, NSS dietary intervention effectively ameliorated these disturbances by reducing the oxidative stress in the serum, reestablishing the glycoprotein and collagen content, and blocking the apoptotic pathway in the kidney.

The absence of significant changes in the kidney functional parameters of the MSG group contradicts the previous findings (Contini et al., 2017; Elbassuoni et al., 2018). However, it should be taken into consideration that the adverse alterations in the renal tissues of rats exposed to MSG, according to our histopathological examination, were of a moderate degree. It seemed that MSG requires a long time to cause end-stage renal diseases resulting in obvious alterations in the kidney function parameters. For instance, glomerular filtration rate, as an index of kidney damage, must be reduced to about 50% before plasma urea and creatinine concentration rise above the upper limit of their respective reference range (Baum et al., 1975). When the kidney is injured, the remaining functional mass responds and attempts to continue to maintain the homeostasis of the internal environment by increasing the perfusion and the size of the remaining glomeruli and increasing the size and function of the remaining tubules (George and Neilson, 2015). Thus, the histopathological abnormalities were not accompanied by loss of renal functional integrity using the current dose and duration of exposure.

The inhibition in SOD activity in the MSG group is due to depletion or inactivation of enzymatic antioxidants by free radicals. This outcome may lead to further generation of superoxide radicals (Singh et al., 2003). Prolonged glutamate toxicity increases the mitochondrial proton gradient due to excessive generation of electron donors by the Krebs cycle, which may subsequently accelerate the production of mitochondrial superoxide anion (Sharma, 2015). Overproduction of reactive oxidants induces α-ketoglutarate dehydrogenase which acts on oxygen to produce superoxide and/or hydrogen peroxide (Tretter and Adam-Vizi, 2005). MSG causes excessive emission of free radicals that initiate and propagate lipid peroxidation cascade (Kayode et al., 2020). All the above factors could be major players in shifting the oxidant/antioxidant balance towards the pro-oxidant side, which in turn exert a compensatory regulatory response by up-regulating the antioxidant defensive mechanism (Chen et al., 2011) represented in this investigation by increased serum TAC. As one member of the phase II detoxification enzymes, GST promotes free radical detoxification and thus plays a leading role in protecting the tissues from oxidative injury (Hayes and Pulford, 1995). Taking into consideration that MSG is a potent oxidative stress inducer, it can be supposed that increased GST activity, as that found in our experimental model, is a defensive response against free radical overproduction (Sharma et al., 2014). Selective inhibition of specific antioxidants and maintenance of the functionality of others resulting in still reasonable defensive mechanisms could be suggested as a potential explanation for the marked reduction in SOD activity in parallel with the enhancement in GST activity and TAC in the current experiment. Actually, the literature is punctuated with some evidence indicating the ability of MSG to activate a wide array of enzymatic and non-enzymatic antioxidants such as glutathione and the enzymes involved in its redox cycle (Choudhary et al., 1996; Onyema et al., 2006). This also explains the positive immuno-expression of GR and SOD2 in the kidney of MSG group, an outcome which is similar to that found in the liver of astaxanthin-challenged rats (Monmeesil et al., 2019). The up-regulation of SOD and other antioxidative enzymes is likely the mechanism to resist the damage from increased oxidative stress (Gulubova and Vlaykova, 2010). GR is essential in maintaining the cellular content of reduced glutathione; one of the most plentiful reducing thiols which play a pivotal role in the control of reactive oxygen species production (Couto et al., 2016). Based on our findings, it appears that oxidative stress caused by MSG exposure induces free radical scavengers such as SOD2 and GR to provide the cells with some resistance against free radical attack.

In our study, the role of NSS in restoring the oxidant/antioxidant equilibrium was evident by the normalization of LPO and TAC similar to earlier studies (Mady et al., 2016; Beheshti et al., 2018). Limitation of reactive oxygen species formation and enhancement of enzymatic and non-enzymatic antioxidants and glutathione redox components (Shahid et al., 2018; Karimi et al., 2019) are the main mechanistic methods by which NSS exhibits antioxidant activity and protects against the adverse effects of food additives. Nevertheless, serum SOD and GST activities in MSG exposed rats did not significantly change in response to NSS supplementation. To resolve this controversy, it should be kept in mind that there are many forms of free radicals that can cause oxidative damage and a wide array of antioxidant defensive molecules that act to restore redox homeostasis (Pisoschi and Pop, 2015). Differential inducibility of anti-oxidant enzymes at both transcriptional and translational levels (Wang and Michaelis, 2010) and differential response to oxidative stress inducers (Mieiro et al., 2010) should also be taken into account. From this perspective, the changes in SOD and GST activities only cannot provide a valid explanation for the ability of NSS to suppress oxidative damage. In this case, TAC is suggested to be the most relevant biomarker for assessing oxidative/reductive potency, taking into account the cumulative synergistic action of all the antioxidants present in the sample, and providing an integrated parameter rather than the simple summation of measurable antioxidants (Ghiselli et al., 2000). On the contrary, the estimation of individual antioxidants may give a misleading picture because antioxidants work in concert through chain-breaking reactions (Brock et al., 2004). Actually, the reduction in TAC points to an improvement in the status of oxidative stress, as the perturbation in redox homeostasis induces up-regulation of endogenous antioxidant defenses, mediated by activation of redox-sensitive transcription factors and its downstream signaling pathways, causing an increase in the overall capacity to antagonize the harmful effects of oxidative damage (Done and Traustadóttir, 2016). In the current study, the down-regulation of SOD2 and GR in hepatic and renal tissues following NSS consumption is in contrast to the up-regulation of enzymatic antioxidants in the liver of hypercholesterolemic rats (Ismail et al., 2010) and cortex and hippocampus of the lead intoxicated mouse (Butt et al., 2018). This controversy may be owing to differences in the type of experimental animal models. The weak immunostaining of SOD2 and GR reflects the free-radical quenching activity of NSS (Butt et al., 2018).

The histopathological alterations in the kidney of the MSG group are in harmony with those previously observed (Elbassuoni et al., 2018). The misbalance in redox circuitry in our study makes the cellular structure of the kidney more vulnerable to peroxidative damage as most of the renal lipids are composed of long-chain polyunsaturated fatty acids (Thomas et al., 2009). High doses of MSG induced long-term ischemia (Bopanna et al., 1999) which cause an increased influx of Ca ions resulting in mitochondrial, lysosomal, and membrane disruption (Allen et al., 1987). MSG triggers bursts of superoxide by increasing mitochondrial proton gradient and glyceraldehyde-3-phosphate dehydrogenase. Superoxide radical is responsible for lipid peroxidation which in turn increases intracellular calcium load and membrane permeability. The swelling of cells caused a drop in aerobic respiration. To maintain the energetic reserve, glycolysis occurs in the cells producing lactate and consequently causing cytoplasmic acidification. This leads to inhibition of sodium-potassium ATPase culminating in membrane damage (Dixit et al., 2014). The unique histo-metabolic characteristics of renal tubular epithelial cells including broad contact surface, high oxygen consumption, and excessive metabolic activity render them particularly sensitive to ischemia and toxins. Also, tubules came in contact with toxic chemicals during their renal handling. The loss of polarity of polarized epithelia of proximal convoluted tubules due to its contact with toxins resulted in their ischemia, then eventual necrosis (Dixit et al., 2014).

Excess collagenous fibers were observed in the renal interstitial tissue in Crossmon’s trichrome-stained sections of the MSG group similar to that reported by other researchers (Sharma et al., 2013). Urolithiasis and oxidative stress due to MSG overintake contribute to fibrosis in the kidney, as reactive oxidants drive the differentiation of fibroblasts to myofibroblasts (Sampson et al., 2011).

Lymphoid cell infiltration in the renal medulla could represent a defensive approach against MSG challenge as lymphocytes secret antitoxins and accelerate cell healing (Curran, 1996). Production of fibrogenic factors as lymphokines by lymphocytes enhances fibroblast proliferation and collagen synthesis (Casini et al., 1985) as indicated by the appearance of thick collagen fibers in the renal structures in the MSG group.

Membranoproliferative glomerulonephritis in the kidney of the MSG group indicates the deposition of immune complexes as a feature of humoral immunity against foreign substances. This leads to the release of chemotactic factors promoting leucocyte accumulation which secrete pro-oxidants and proteases mediating capillary wall injury, proteinuria, and impaired glomerular filtration rate (Sethi et al., 2019). Accumulation of macrophages within the renal interstitial spaces, induction of heme-oxygenase, and the presence of a pro-oxidant environment (Agarwal et al., 1996; Vielhauer et al., 2001) could be responsible for the renal tubular alterations following MSG exposure.

Decreased PAS positivity in the kidney of the MSG group is similar to that found in earlier studies (Moussa and Al Mulhim, 2013; Elbassuoni et al., 2018) giving an indication of decreased glycoprotein accumulation. MSG has deteriorated consequences on carbohydrate metabolism by decreasing glucose utilization through glycolysis, accelerating hepatic glucose liberation to the bloodstream, and depleting glycogen storage (Diniz et al., 2004). On the other hand, NSS restored tissue glycogen storage owing to the stimulation of pancreatic insulin secretion which activates glycogen synthase (Abdelrazek et al., 2018).

The obvious improvement in renal histoarchitecture of MSG challenged rats following NSS administration is in the same line as other investigators (Moussa and Al Mulhim, 2013). Thymoquinone confers renal cytoprotection against food additives by reducing renal damage biomarkers, lipid peroxidation, and activating enzymatic antioxidants (Al-Seeni et al., 2018). Antioxidant, anti-inflammatory, and anti-apoptotic characteristics of thymoquinone along with the ability of NS to act as a vasodilator to the renal microvasculature (Mahmoud et al., 2014; Purnamayanti et al., 2018) are implicated in normalizing renal histological patterns and protecting the glomeruli and renal tubules from degenerative and necrotizing chemotoxicants.

The attenuation of lymphoid cell infiltration in the renal medulla of the MSG + NSS group reflects the ability of NSS as a potent immunemodulator (Niu et al., 2021) and its significant role in enhancing of cellular immunity (Ebaid et al., 2011). It decreases the migration of these cells from the peripheral blood because it has an anti-inflammatory action (Houghton et al., 1995) by suppressing the production of tumor necrosis factor alpha and interleukin 6 (Jaswal, et al., 2022) and decreasing the transcription level of cyclooxygenase-2 (Al Wafai, 2013). Thymoquinone downregulates the gene expression of chemoattractant cytokines (Taka et al., 2015) and prevents biosynthesis of inflammatory mediators (Shaterzadeh-Yazdi et al., 2018).

The pro-apoptotic effect of MSG on the renal tissues is in harmony with that observed in the testicular tissue of rats (Abd-Elkareem et al., 2021). MSG induces a marked elevation in pro-apoptotic proteins such as caspase-3, P53 and Bax together with a reduction in anti-apoptotic ones including Bcl-2 (Shukry et al., 2020). The increase in glutamate secondary to consumption of MSG encouraged Ca^2+^ inflow and disturbance of the inner mitochondrial transmembrane potential, which resulted in opening the mitochondria permeability transition pore (Khodorov et al., 2002; Kanki et al., 2004). When permeability transition pore is unchecked, multiple essential signaling molecules come into play to initiate apoptosis (Desagher and Martinou, 2000). As an inducer of oxidative stress, MSG can cause cell death by prompting the intrinsic and extrinsic apoptotic pathways (Sarhan, 2018). The obvious reduction in the number of apoptotic cells in the kidney when NSS was supplemented in parallel with MSG is corresponding to that reported by (Mousavi and Mohajeri, 2014) and can be attributed to its antioxidant, immunomodulatory and genoprotective effects (Babazadeh et al., 2012). Similarly, NS oil decreased Bax/Bcl2 levels and down-regulated the expressions of caspase-3 (pro and cleaved forms) proteins in the brain and liver tissues of mice after d-galactose-induced aging (Shahroudi et al., 2017). Thymoquinone suppressed caspase-3 activity and down-regulated the transcript level of caspase-3 in isolated rat heart following ischemia reperfusion injury (Xiao et al., 2018). Squalene acts as a DNA-damage checkpoint inhibitor through intracellular induction of Wip1 expression (Tatewaki et al., 2016). The decrease in the levels of Bax and cleaved caspase-3, and the increase in the level of Bcl-2 underlie the ability of linolenic acid to block the apoptosis in cisplatin-induced renal intoxicated mice (İstifli et al., 2019).

## Conclusion

MSG-challenged rats were characterized by serum redox imbalance and increased programmed cell death along with renal dysfunction and histochemical impairments, but the deteriorations in the histochemical features of the kidney did not reflect on its functional parameters. The negative consequences of MSG consumption were partially overcome by the nutritional inclusion of NSS by restoring the redox potential and ameliorating the histopathological deteriorations, decreased glycoprotein, fibrosis and apoptosis in the kidney. These outcomes are of significant importance in paving the road towards the incorporation of NSS as a candidate strategy against MSG-induced abnormalities, and opening interesting possibilities for studying its effectiveness in fighting other side effects of MSG.

According to our findings, NSS provided a novel and effective strategy for the prevention of MSG-induced renal damage especially on the levels of cytoarchitecture and programmed cell death before the renal pathogenic biomarkers can be altered. These findings are of significance in directing the attention towards the incorporation of NSS as a promising natural approach to offset the MSG-associated nephrotoxicity, and opening interesting possibilities for studying its usefulness in combating the other consequences of MSG. However, one of the limitations of the present study is that it does not cover the reno-protective mechanisms of NSS at the molecular levels, therefore we warranted to explore these issues in depth in further studies.

## Data Availability

The original contributions presented in the study are included in the article/Supplementary Materials, further inquiries can be directed to the corresponding author.

## Abbreviations

GR, glutathione reductase; GST, Glutathione-S-transferase; LPO, lipid peroxides; MSG, monosodium glutamate; NO, nitric oxide; NSS, Nigella sativa L. seed; SOD, superoxide dismutase; TAC, total antioxidant capacity.
